# Invasive Assessment of the Myocardial Microcirculation during Beating Heart Coronary Artery Bypass Grafting

**DOI:** 10.3390/jcm9030663

**Published:** 2020-03-01

**Authors:** Marcin Hellmann, Jakub Piotrowski, Mariusz Kaszubowski, Maria Dudziak, Lech Anisimowicz

**Affiliations:** 1 Department of Cardiac Diagnostics, Medical University, 80-214 Gdansk, Poland; mdudziak@gumed.edu.pl; 2 Department of Cardiac Surgery, University Hospital, 85-094 Bydgoszcz, Poland; piotrowski.jakub1@gmail.com (J.P.); lech.anisimowicz@gmail.com (L.A.); 3 Institute of Statistics, Department of Economic Sciences, Faculty of Management and Economics, University of Technology, 80-233 Gdansk, Poland; mkaszubo@zie.pg.gda.pl

**Keywords:** myocardial microcirculation, coronary artery bypass grafting, laser Doppler, microvascular perfusion monitoring, coronary collateral circulation

## Abstract

Coronary artery bypass grafting may be associated with several cardiac complications, including ischemia, acute myocardial infarction, arrhythmias, or hemodynamic instability. Accumulating evidence suggests that well-developed coronary collateral circulation may protect against adverse effects, including myocardial ischemia. Assessment of myocardial microvascular perfusion is, therefore, of great clinical interest in beating heart surgery. In this paper, myocardial microvascular perfusion is continuously assessed on the beating heart using laser Doppler flowmetry in consecutive patients who underwent coronary artery bypass grafting procedures. No significant (*p* = 0.110) differences were found between the averaged perfusion signal (*n* = 42) at the baseline, during artery occlusion, or after reperfusion (732.4 ± 148.0 vs. 711.4 ± 144.1 vs. 737.0 ± 141.2, respectively). In contrast, significantly different (*p* < 0.001) mean perfusion signals (*n* = 12) were found (805.4 ± 200.1 vs. 577.2 ± 212.8 vs. 649.3 ± 220.8) in a subset of patients who presented with hemodynamic instability and myocardial ischemia. Additionally, a strong positive correlation between the plasma levels of high-sensitivity troponin I and perfusion decrease level after artery occlusion was found (*r* = 0.854, *p* < 0.001). This study argues that myocardial microvascular perfusion remains constant during coronary artery bypass grafting on the beating heart in advanced coronary artery disease. This phenomenon is most likely due to an extensive coronary collateral circulation.

## 1. Introduction

Coronary artery disease (CAD) is the single largest cause of death in industrialized countries. There is compelling evidence for a link between microvascular dysfunction, coronary collateral development, and the pathogenesis of CAD. Coronary artery bypass grafting (CABG) surgery and percutaneous coronary intervention (PCI) are both well-established revascularization methods to treat CAD. CABG is associated with the reduction of mortality, and remains a standard therapy in patients with extensive CAD when compared with PCI and pharmacological treatment alone [1].

CABG with the use of cardiopulmonary bypass (CPB) is recognized as the “gold standard” technique in terms of safety and effectiveness for surgical myocardial revascularization. A further effort in minimizing the occurrence of some complications related to conventional CABG has led to the development of off-pump coronary artery bypass (OPCAB), a technique in which the anastomoses are performed on the beating heart. Taken together, accumulating evidence suggests that OPCAB may significantly reduce the rate of mortality and morbidity when compared with conventional CABG [2].

One of the worst potential complications of coronary artery bypass graft surgery could be early graft failure causing myocardial ischemia or acute myocardial infarction. However, CABG surgery could be associated with several other cardiac complications, including arrhythmias or hemodynamic instability. Therefore, the possibility of intraoperative and postoperative myocardial blood flow assessment is of major clinical importance, especially in vascular surgery [3]. Several studies have indicated that laser Doppler flowmetry (LDF) is able to detect the real-time fluctuations in myocardial perfusion under different circumstances [4]. Of note, there is relatively little data concerning myocardial microvascular measurements in humans, including CABG surgery on the beating heart. 

Thus, the assessment of the myocardial microvascular perfusion is of great interest in various experimental and clinical studies. Accumulating evidence suggests that coronary collaterals may protect against myocardial ischemia and other secondary cardiac complications, and their presence is associated with improved survival in patients with coronary artery disease [5]. However, conducting a precise and complex clinical assessment of coronary collateral circulation is a great challenge. 

We recently showed a clinical image of a patient with extensive CAD undergoing off-pump coronary artery bypass grafting, where microvascular flow remained constant during the whole procedure. We hypothesized that it could be due to well-established collateral circulation, which was exceptionally well-illustrated on the angiogram [6]. Thus, the aim of this study is to investigate the mechanism of this phenomenon in a larger group undergoing CABG as well as to better understand the behavior of the myocardial microcirculation during CABG on the beating heart.

## 2. Materials and Methods

### 2.1. Study Population

This study enrolled twenty-six consecutive patients with extensive coronary artery disease who qualified for coronary artery bypass grafting. All patients were treated before and after surgery according to the current European Society of Cardiology Guidelines. Twenty-five participants were operated upon without a support of extracorporeal circulation. In one case, on-pump beating heart CABG procedure had to be performed. Ten patients were admitted to the hospital with a diagnosis of myocardial infarction. In one case, PCI procedure was performed before CABG. Fifteen patients were admitted to the hospital with stable coronary artery disease; one patient was operated upon immediately after admission due to hemodynamic instability, and other patients were clinically stable and operated upon according to the schedule.

From November 2018 through April 2019, 26 patients underwent coronary artery surgery through a median sternotomy. The mean age was 64.9 ± 7.2 years. There were 6 women and 20 men. Fifty-four anastomoses were performed during off-pump coronary artery surgery.

The study conforms to the principles outlined in the Declaration of Helsinki. The study protocol was approved in October 2018 by the Independent Ethics Committee at the Nicolaus Copernicus University (IRB no. 703). All subjects gave written informed consent before participation.

### 2.2. Operative Technique

Anesthetic procedures were standardized for all patients. To exclude the bias of a surgeon’s experience and preference, all surgical procedures were performed by one surgeon who had been specifically trained in off-pump coronary heart surgery. All interventions were performed in a typical way via a midline sternotomy. The choice of conduits was based on the surgeon’s preference as well as being dictated by patient characteristics. All patients received two or more grafts using various combinations of left and/or right internal mammary artery and/or saphenous vein grafts. The myocardial tissue stabilizer (Octopus Tissue Stabilization System, Medtronic, USA) was applied to immobilize the target site of coronary anastomosis.

### 2.3. Laser Doppler Myocardial Microvascular Perfusion Measurements during CABG

**Figure 1:**
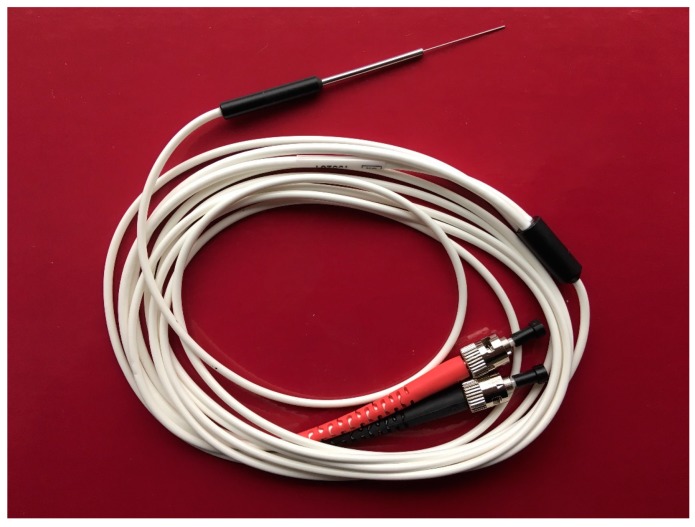
Laser Doppler intramuscular fiber-optic perfusion probe.

**Figure 2:**
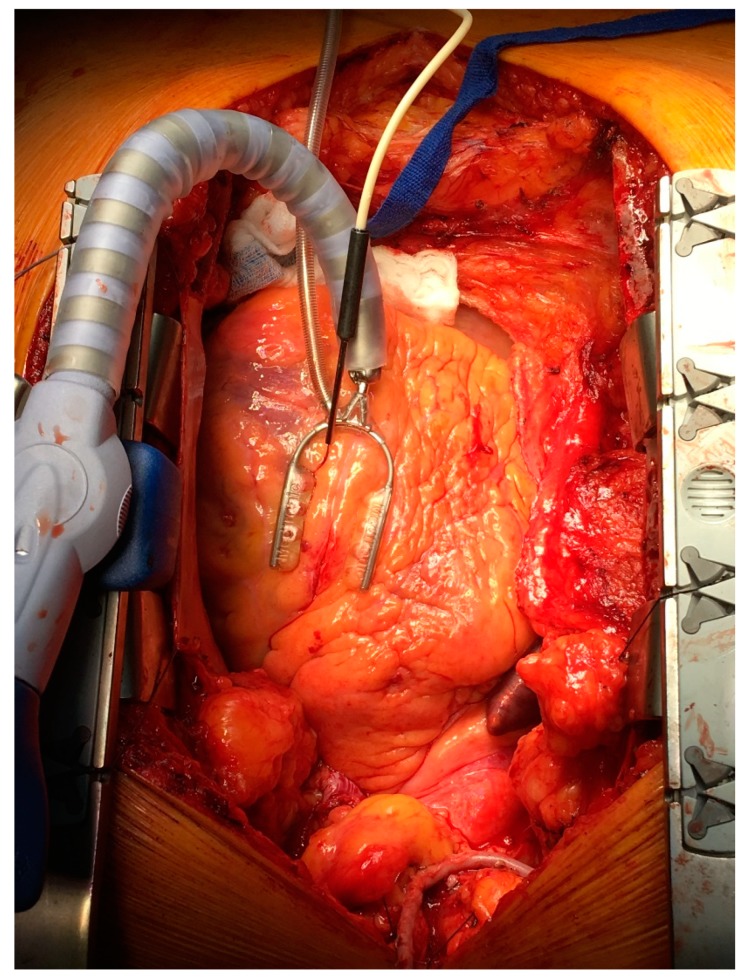
Laser Doppler intramuscular fiber-optic perfusion probe was inserted into the myocardium during beating heart surgery downstream from the planned anastomosis.

All measurements of myocardial microvascular perfusion were carried out using laser Doppler flowmetry (Periflux System 5000, Perimed, Järfälla, Sweden) equipped with the special insertion probe (Stainless Steel Probe 411-311, Perimed, Järfälla, Sweden). Laser Doppler intramuscular fiber-optic perfusion probe (Figure 1) was inserted 3–5 mm into the myocardium during beating heart surgery downstream from the planned anastomosis (Figure 2). After clamping and incision of coronary artery, an anastomosis was performed with prepared graft. When anastomosis was completed, clamps were taken off and the flow through the coronary artery was restored. All measurements were carried out continuously during the operation. Baseline registration was performed for approximately 3 min. Then, the perfusion measurements were continued during vascular anastomosis and approximately 3 min after occlusion release. At the end, the needle probe was removed from the myocardium and subsequently the next anastomosis was performed.

The Laser Doppler flowmetry technique is based on the emission of a beam of laser light carried by a fiber-optic probe. Tissue was illuminated with coherent laser light of 780 nm from a laser diode through a fiber optic light guide. Backscattered light was registered using the same probe, and frequency-shifted light was extracted using the heterodyne light-beating technique. All measurements were expressed as arbitrary perfusion units (PU) [7]. 

### 2.4. Transit Time Flowmetry Measurements

The principle of transit time flowmetry measurements (TTFM) is based on registering the difference between upstream and downstream transit time of a wide ultrasound beam. The transit time difference is directly proportional to the blood volume flow. This measurement principle gives an accurate quantification of the real time volume flow that compliments the Doppler principle’s velocity measurement (mL/min). All measurements of the flow through the arterial grafts were carried out using TTFM (VeriQ, Medistim, Oslo, Norway).

### 2.5. Cardiac Troponin Testing

Blood samples were obtained from a venous puncture immediately after the operation every 12 h until the highest level was reached. The highest level of high-sensitivity troponin I (hs-TnI) was used for analyses. Plasma levels of hs-TnI were measured by standard techniques using commercially available assay (Abbott Laboratories, Abbott Park, IL, USA). This assay has a limit of detection between 1.2 ng/L and 1.9 ng/L, and the interassay coefficient of variation is <10% at 4.7 ng/L. The upper reference limit, or 99th-centile value, is 34 ng/L in men.

### 2.6. Data Analysis

Data were digitized, stored on a computer, and analyzed offline with signal processing software (PeriSoft 2.5.5, Perimed, Järfälla, Sweden). Myocardial microvascular blood flow was expressed as perfusion units (PU), averaged over 3 min at baseline during intraoperative artery occlusion, and averaged over 3 min after release of occlusion. Fifty-four coronary anastomoses were performed. Patients were divided into two groups based on significant percentage decreases in perfusion signal between baseline and perfusion values after coronary artery clamping. A perfusion signal drop greater than 15% was considered statistically significant.

### 2.7. Statistical Analysis

Quantitative data are expressed as the mean and standard deviation. Frame chart with mean values were improved with 95% confidence intervals. Normality assumption was verified with Shapiro-Wilk test or Q-Q plots. Differences between mean values of perfusion were examined by parametric ANOVA for dependent variables and post-hoc Tukey HSD (Honest Significant Difference) test. To assess the interdependence between the analyzed variables, Pearson’s correlation coefficients were calculated and regression lines were presented with appropriate scatter plots. In the case of nonlinear correlations, an additional nonparametric Spearman’s rank correlation coefficient was designated. A *p*-value of < 0.05 was considered statistically significant. All statistical analyses were performed with Statistica version 13.1 (Dell Inc. 2016, data analysis software system).

## 3. Results

### 3.1. Patient Characteristics 

The demographic and clinical characteristic of the study group are summarized in Table 1. The majority of patients presented with a good ejection fraction. All participants completed the protocol. Fifty-four coronary anastomoses were performed. The bypass characteristics are summarized in Table 2. Patients were divided into two groups based on significant percentage decrease in perfusion signal between baseline and perfusion values after coronary artery clamping.

### 3.2. Perfusion Signal Analyses on the Beating Heart

**Figure 3:**
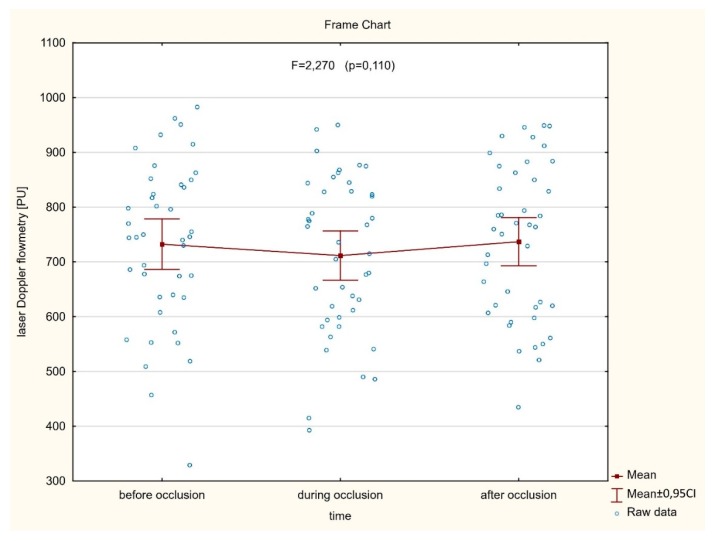
There are no significant differences between averaged myocardial microvascular perfusion signal at the baseline, during artery occlusion, and after reperfusion, assessed continuously on the beating heart during coronary artery bypass grafting (CABG) procedures.

**Figure 4:**
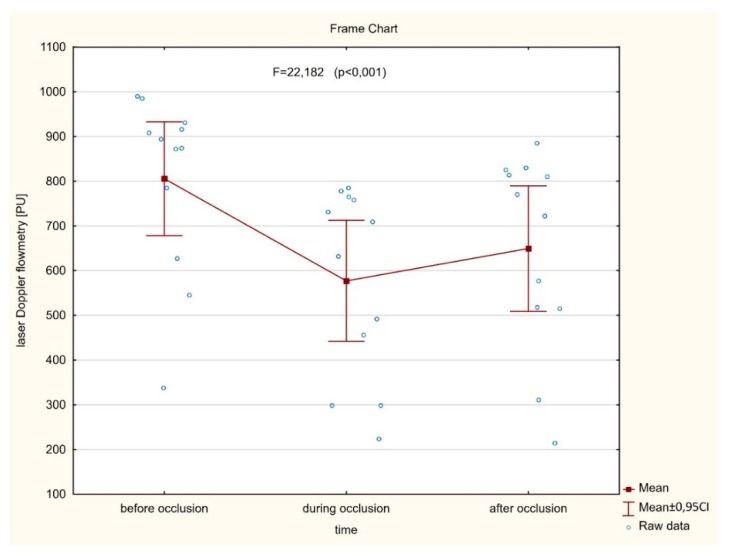
A significant difference between averaged myocardial microvascular perfusion signal at the baseline, during artery occlusion, and after reperfusion assessed continuously on the beating heart during CABG procedures in subset of patients presented with hemodynamic instability and myocardial ischemia.

As shown in Figure 3 (*n* = 42), no significant (*p* = 0.110) differences were found between the averaged perfusion signal at the baseline, during artery occlusion, or after reperfusion (732.4 ± 148.0 vs. 711.4 ± 144.1 vs. 737.0 ± 141.2, respectively). In contrast, there was a significant decrease of the perfusion signal in only six cases where LIMA was grafted to the diagonal branch. In the other six cases, with an important perfusion drop, patients presented with clinical symptoms of hemodynamic instability or myocardial ischemia during procedures. In a subset of twelve cases mentioned above, significantly different (*p* < 0.001) mean perfusion signals were found (805.4 ± 200.1 vs. 577.2 ± 212.8 vs. 649.3 ± 220.8) (Figure 4). More precisely, there was a statistically significant decrease of the perfusion signal at the baseline vs. artery occlusion (*p* < 0.001), and likewise between baseline and reperfusion (*p* = 0.001), but there were no significant differences (*p* = 0.112) between mean values of perfusion during artery occlusion and after reperfusion. However, an important trend was found towards the increase of the perfusion after the end of the artery clamping. 

### 3.3. Correlation Analyses

**Figure 5:**
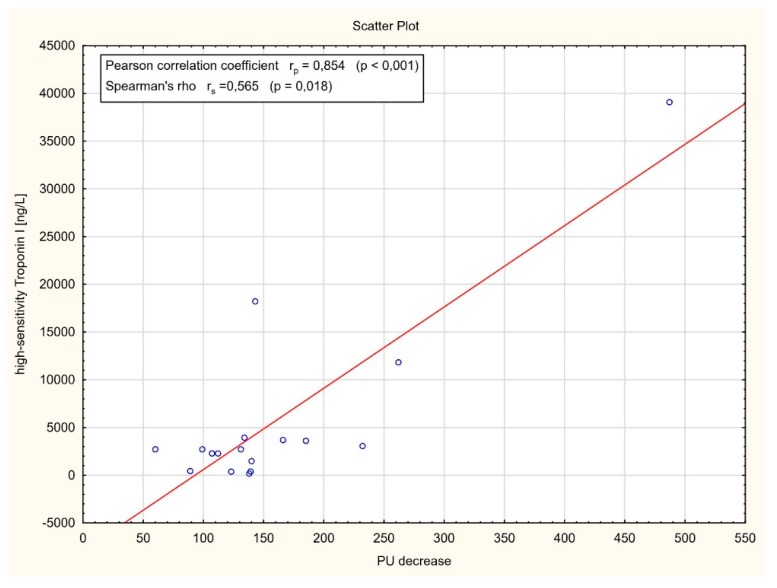
Correlation analysis between the plasma levels of high-sensitivity Troponin I (hs-TnI) and laser Doppler flowmetry (LDF) perfusion units (PU) decrease after artery occlusion.

**Figure 6:**
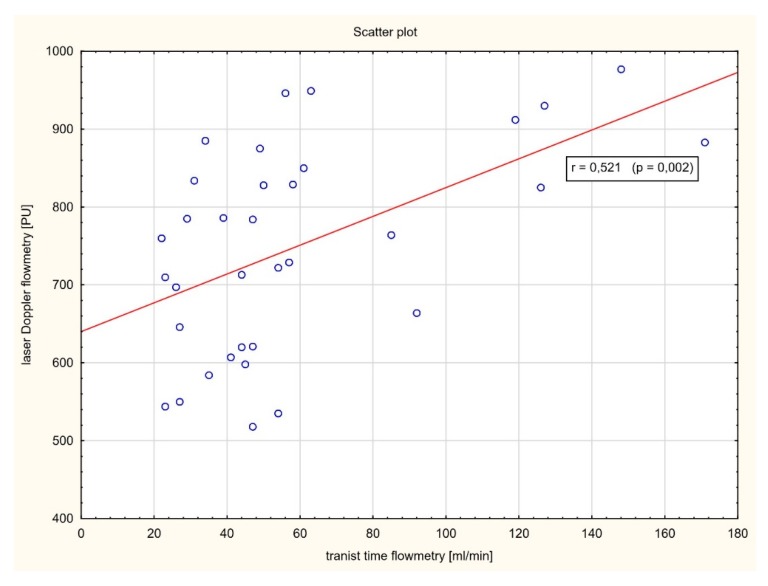
Correlation analysis between myocardial perfusion after CABG assessed by LDF and blood flow in the in coronary bypass grafts measured by transit time flowmetry (TTF).

As shown in Figure 5, a strong positive correlation between the plasma levels of high-sensitivity Troponin I and perfusion decrease level after artery occlusion was found (*r* = 0.854, *p* < 0.001). Similarly, a significant positive correlation between myocardial perfusion after CABG assessed by laser Doppler flowmetry and blood flow in the in coronary bypass grafts measured by transit time flowmetry was observed (*r* = 0.521, *p* = 0.002) (Figure 6).

### 3.4. Safety of the Laser Doppler Procedure 

We also assessed the safety of the procedure concerning intraoperative laser Doppler measurements. As mentioned above, intramuscular fiber-optic perfusion probe was inserted 3–5 mm into the myocardium during beating heart surgery downstream from the planned anastomosis. All procedures were well-tolerated. There were no bleeding complications. We did not observe any significant drop in blood pressure. In one case, the insertion of the probe into the myocardium was associated with arrhythmia presented as ventricular extrasystoles.

## 4. Discussion

The main purpose of this study was to assess the influence of coronary artery bypass grafting procedures on heart microcirculation. To our knowledge, our results demonstrate for the first time that myocardial microvascular perfusion remains constant during CABG on the beating heart in the vast majority of human patients with extensive coronary artery disease. In this study, laser Doppler flowmetry, a well-established method for microvascular measurements, was used to assess myocardial perfusion during all procedures. Of note, there are few papers regarding LDF perfusion measurements on the beating heart and most of them concern animal studies. 

The key finding of this study is that myocardial microvascular blood flow assessed continuously did not change after clamping of the artery compared with the baseline flow. Similarly, the perfusion remained constant after the end of the artery occlusion. According to our assumption, this phenomenon is most likely to be due to an extensive collateral circulation occurring in the operated patients with advanced coronary artery disease. Indeed, we found well-established collateral circulation on many angiograms performed prior to the procedures, although it was not the aim of this study. Accumulating evidence suggests that well-developed coronary anastomoses originating from a collateral supplying artery can entirely compensate for the occlusion in the collateral-receiving vessel. However, the amplitude of myocardial ischemia during coronary occlusion is not only dependent on collateral circulation but also on other aspects, including the occlusion period; the size of the ischemic territory; the occurrence of preconditioning episodes of ischemia; and the actual level of oxygen consumption, which itself is determined by myocardial contractility, heart rate, and blood pressure [8].

In this study, we performed 54 CABG procedures on the beating heart. The majority of the anastomoses were performed with arterial grafts. Most of them involved the left internal mammary artery (LIMA) to left anterior descending coronary artery (LAD) graft, which is the most common anastomosis in CABG surgery. We did not observe any important drop or change in the perfusion signal during those procedures. However, we found a significant decrease of the myocardial microvascular blood flow while performing LIMA bypass graft to the diagonal branch in six cases. These results led us to the presumption that myocardial territory supplied by diagonal branch is not collateralized as well as other areas. Additionally, we found a significantly lower perfusion after artery occlusion when patients presented with clinical symptoms of hemodynamic instability or myocardial ischemia during procedures. Of note, three situations concerned left anterior descending and another three marginal branch clamping.

Coronary collateral circulation is a network of the small vessels that allow the connection between the different parts of an epicardial vessels. Taken together, accumulating evidence suggests that well-developed collateral circulation contributes positively to the course of coronary artery disease. Recently, a large body of evidence has indicated that the presence of an extensive collateral circulation has been associated with the decrease of myocardial infarction in patients with stable CAD [9]. The most widely used invasive method for assessing coronary collateral circulation is contrast angiography. However, it does not provide information regarding the vascular and tissue response that occur during ischemia. In parallel to the new approaches in heart surgery, including surgery on the beating heart, there is a need to develop methods for myocardial microvascular perfusion measurements, both in association with bypass grafting and postoperatively in the intensive care unit. In our study, myocardial blood flow was measured using laser Doppler flowmetry, which enables the evaluation of perfusion in a small tissue volume. Skin microcirculation is routinely studied in humans by using noninvasive laser Doppler flowmetry [10,11]. However, this technique was very rarely used in different vascular beds such as the heart, while the assessment of the myocardial microvascular perfusion is of significant interest in various experimental and clinical studies. To our knowledge, in 1988, Ahn et al. assessed the myocardial perfusion on the beating pig’s heart using LDF for the first time [12]. In another experimental animal model, Bierbach et al. assessed the behavior of myocardial microcirculation using LDF after controlled reduction of LAD blood flow to 70% and 30% of baseline in a healthy porcine model. A statistically significant reduction in myocardial perfusion was found when LAD blood flow was reduced by 70% and when LAD was totally closed. In one animal, coronary occlusion at the end of the experiment only led to a 50% reduction in epicardial myocardial blood flow compared with the baseline level. After sacrifice of this animal and dissection of the myocardial specimen, an intramyocardial ramus intermedius was found supplying the lateral aspect of the left ventricle’s anterior wall close to the observed area [13]. Karlsson et al. assessed the myocardial microvascular perfusion signal by LDF in a small group of patients (*n* = 13) during CABG. A significantly lower perfusion signal in the arrested heart, compared with the beating heart, was registered. Additionally, no significant difference between pre- and post-CABG was found [14]. Such data are consistent with our report. However, in our study, microvascular perfusion signal was assessed continuously and also registered during the procedure.

Myocardial revascularization in patients with left ventricular dysfunction is performed to decrease mortality, reduce the symptoms, and prevent future ischemic events. In patients with CAD, a well-developed coronary collateral circulation is related to reduction of infarct size, left ventricule dysfunction, and all-cause mortality. Thus, the clinical profits of coronary revascularization should be carefully considered against the risk of reducing the protective support derived from coronary collateral circulation [5]. As previously mentioned, our data suggest that collateral coronary circulation is efficient enough to maintain myocardial perfusion during coronary artery grafting procedures on a beating heart in patients with advanced coronary artery disease. However, our results have no purpose to undermine the idea of well-established surgical revascularization methods but rather open the discussion about careful indications to CABG procedures according to new pathophysiological findings. 

Another issue is myocardial cell injury, which is an unavoidable process during heart surgery, leading to the increase of various cardiac biomarkers, including the troponins [15]. In this study, after coronary artery clamping, troponin I levels significantly increased. We found that an important decrease of the perfusion signal is positively related to the increase in plasma level of high-sensitivity troponin I. However, the troponin release profile could be affected by many factors, including insufficient myocardial protection, embolism, local or global ischemia, and surgical procedure injuries. 

In clinical practice, most studies focused on the assessment of coronary blood flow. In this study, we were able to measure simultaneously myocardial perfusion after CABG using laser Doppler flowmetry and blood flow in the in coronary bypass grafts measured by transit time flowmetry. Our data demonstrate for the first time in humans a significant positive correlation between myocardial microvascular perfusion and blood flow in the coronary arterial grafts. One group assessed the myocardial perfusion in the beating pig’s heart using LDF [12]. Both epicardial and intramuscular perfusion measurements were performed continuously, and laser Doppler perfusion signal was found to correlate well with coronary blood flow. Such data are consistent with another report showing that coronary artery flow measured by TTFM is related to regional endocardial perfusion in the corresponding myocardial area in animal model [13].

This study has several limitations. First, the study is limited by the relatively small cohort of patients, which is a result of invasiveness of the procedure and the highly selective enrollment process to achieve the best quality of measurements during beating heart surgery. It should also be stressed that the installation of the equipment and the laser Doppler measurements extend the duration of standard CABG procedure. Still, it is the first study of its kind performed with humans.

## 5. Conclusions

In conclusion, this study argues that myocardial microvascular perfusion remains constant during coronary artery bypass grafting on the beating heart in advanced coronary artery disease. Indeed, further mechanistic studies are needed to explain the complex regulation mechanisms of myocardial microcirculation. Data from this study can be helpful to design multicentric clinical trials with larger sample sizes. Additionally, our study shows that insertion of the perfusion probe into the myocardium during CABG procedure is safe, well-tolerated, and not associated with any significant cardiac adverse effects. Invasive laser Doppler measurements are feasible, reproducible, and sensitive to acute changes in myocardial microvascular perfusion in humans. This device would be highly useful in predicting myocardial ischemia in patients undergoing cardiac surgery.

## Figures and Tables

**Table 1:** Patient characteristics.

Male/Female (*n*/*n*)	20/6
Age (years), mean (SD)	64.9 (7.2)
Hypertension, *n* (%)	17 (65)
Diabetes, *n* (%)	9 (35)
Dyslipidemia, *n* (%)	14 (54)
COPD, *n* (%)	2 (8)
PAVK, *n* (%)	3 (12)
Previous MI, *n* (%)	10 (38)
Ejection fraction (%), *n* (%)	
>50	20 (77)
30–49	5 (19)
<29	1 (4)
BITA	10

BITA: bilateral internal thoracic arteries; COPD: chronic obstructive pulmonary disease; PAVK: peripheral arterial occlusive disease.

**Table 2:** Distribution of target vessels and grafts.

	Number	%
**Arterial**		
LIMA – LAD	15	27.8
RIMA – LAD	10	18.5
LIMA – Marg	9	16.7
LIMA – Diag	5	9.3
RIMA – Marg	1	1.9
**Venous**		
VSM – Marg	5	9.3
VSM – IM	4	7.4
VSM – Diag	2	3.7
VSM – PDA	1	1.9
VSM – PLCx	1	1.9
VSM – PLRCA	1	1.9

Anastomosis: LIMA: left internal mammary artery; RIMA: right internal mammary artery; LAD: left anterior descending artery; Marg: marginal branch; VSM: great saphenous vein; Diag: diagonal branch; IM: intermediate artery; PDA: posterior descending artery; PLCx: posterolateral branch of circumflex artery; PLRCA: posterolateral branch of right coronary artery.
